# HB-EGF Ameliorates Oxidative Stress-Mediated Uterine Decidualization Damage

**DOI:** 10.1155/2019/6170936

**Published:** 2019-12-02

**Authors:** Hai-Fan Yu, Cui-Cui Duan, Zhan-Qing Yang, Yu-Si Wang, Zhan-Peng Yue, Bin Guo

**Affiliations:** ^1^College of Veterinary Medicine, Jilin University, Changchun, China; ^2^Institute of Agro-Food Technology, Jilin Academy of Agricultural Sciences, Changchun, China

## Abstract

HB-EGF is essential for uterine decidualization, but its antioxidant function remains largely unclear. Here, we found that HB-EGF promoted the proliferation of stromal cells followed by the accelerated transition of the cell cycle from G1 to S phase and enhanced the expression or activity of Prl8a2, Prl3c1, and ALP which were well-established markers for uterine stromal cell differentiation during decidualization. Under oxidative stress, stromal cell differentiation was impaired, but this impairment was abrogated by rHB-EGF accompanied with the reduced levels of ROS and MDA which were regarded as the biomarkers for oxidative stress, indicating an antioxidant role of HB-EGF. Further analysis revealed that HB-EGF enhanced the activities of antioxidant enzymes SOD, CAT, and GPX, where addition of GPX inhibitor MS attenuated the induction of rHB-EGF on Prl8a2, Prl3c1, and ALP. Meanwhile, HB-EGF rescued the content of GSH and restored the ratio of GSH/GSSG after exposure to H_2_O_2_ but did not alter NOX activity. Along with a decline for mitochondrial superoxide, exogenous rHB-EGF improved the damage of oxidative stress on mtDNA copy number, ATP level, mitochondrial membrane potential, and activities of mitochondrial respiratory chain complex I and III whose blockage by ROT and AA led to a failure of rHB-EGF in protecting stromal cell differentiation against injury. Moreover, HB-EGF prevented stromal cell apoptosis by inhibiting Caspase-3 activity and Bax expression and recovering the level of Bcl-2 mRNA. Collectively, HB-EGF might ameliorate oxidative stress-mediated uterine decidualization damage.

## 1. Introduction

Uterine decidualization, which involves extensive proliferation and differentiation of stromal cells, is essential for placentation and maintenance of successful pregnancy, because its impairment leads to varieties of pregnancy disorders, such as embryo miscarriage and early pregnancy loss [1–3]. Among many factors affecting decidualization, oxidative stress has received much attention and is defined as the increase of reactive oxygen species (ROS) including superoxide (O_2_^−^), hydrogen peroxide (H_2_O_2_) and hydroxyl radical (OH^−^), which are generated as by-products of aerobic respiration and metabolism [4, 5]. Accumulated evidence has revealed that oxidative stress impairs the decidualization process accompanied by an induction of stromal cell apoptosis and lessens the number of implantation sites [6–8]. Meanwhile, excessive ROS resulted in a spectrum of female reproductive disorders including recurrent miscarriage, preeclampsia, and endometriosis [4, 5].

Heparin-binding EGF-like growth factor (HB-EGF), a member of the epidermal growth factor (EGF) family, was highly expressed in uterine luminal epithelium and decidua and crucial for embryo implantation, decidualization, and pregnancy [9–11]. Conditional deletion of uterine HB-EGF resulted in the deferral of implantation window with compromised litter size [11]. Meanwhile, HB-EGF might induce stromal cell differentiation during in vitro decidualization [9, 10]. Although it has been previously reported that HB-EGF decreased ROS production in the leukocytes and intestinal epithelial cells as well as in injured intestine [12], its role in oxidative stress-mediated uterine decidualization damage remains to be established.

The present study is aimed at investigating the effects of HB-EGF on ROS generation, antioxidant enzyme activity, mitochondrial function, and cell apoptosis in H_2_O_2_-induced uterine stromal cells. These results revealed that HB-EGF might protect uterine stromal cell differentiation from oxidative stress.

## 2. Materials and Methods

### 2.1. Animal

Matured Kunming white strain mice (6-9 weeks old) were housed in the animal care facility at the College of Veterinary Medicine, Jilin University, according to the institutional guidelines for the care and use of laboratory animals. Female mice were mated with fertile males of the same strain to induce pregnancy by COC aging (day 1 = day of vaginal plug). All animal procedures were approved by the Institutional Animal Care and Use Committee of Jilin University.

### 2.2. Isolation and Treatment of Uterine Stromal Cells

Uterine stromal cells from day 4 of pregnancy were isolated by enzymatic digestion as previously described [13] and induced for in vitro decidualization with fresh medium supplemented with estradiol-17*β* (Sigma, E1024, 10 nM) and progesterone (Sigma, 850454, 1 *μ*M) in DMEM-F12 with 2% charcoal-treated FBS (Gibco, 12676-029).

After treatment with recombinant human HB-EGF protein (rHB-EGF, R&D Systems, 259-HE, 100 ng/ml), stromal cells were subjected to in vitro decidualization. For further studies, stromal cells were treated as described above and then exposed to 100 *μ*M H_2_O_2_ for 4 h in the absence or presence of ROS scavenger N-acetyl-L-cysteine (NAC, Beyotime, S0017, 5 mM), GPX inhibitor Mercaptosuccinic Acid (MS, Sigma, V900745, 300 *μ*M), mitochondrial respiratory chain complex I inhibitor Rotenone (ROT, MCE, HY-B1756, 2 *μ*M), and mitochondrial respiratory chain complex III inhibitor Antimycin A (AA, Abcam, ab141904, 2 *μ*M). Progesterone, estradiol-17*β*, AA, and ROT were dissolved in ethanol or DMSO, while rHB-EGF, NAC, and MS were dissolved in PBS. Controls received the vehicle only.

### 2.3. Real-Time PCR

Total RNAs were isolated using TriPure reagent (Roche, 11667165001) and reverse-transcribed into cDNA which was amplified using FS Universal SYBR Green Real Master (Roche, 06924204001) to determine the expression level of different genes. After analysis using the 2^-*ΔΔ*Ct^ method, data were normalized to Gapdh expression. The primer sequences used for real-time PCR are listed in Table 1.

### 2.4. ELISA

After different treatments, culture supernatant was collected and centrifuged for 10 min. Concentration of HB-EGF protein was measured using commercial ELISA kits (JiangLai Biotechnology, JL20342). The intra- and interassay coefficient of variation for this assay was less than 11%, and standard curve ranged from 12.5 to 400 pg/ml. Briefly, 50 *μ*l of supernatant or standard was incubated with 100 *μ*l horseradish peroxidase-conjugated antibody for 60 min. After washing with buffer, 100 *μ*l of substrate mixture was added into each well and incubated for 15 min followed by a supplementation of stop solution. Absorbance was measured at 450 nm, and protein concentration was calculated according to curve equation.

### 2.5. RNA Interference

Transfection for siRNA was performed according to Lipofectamine™ 3000 protocol (Invitrogen, 2067544). After transfection with HB-EGF siRNA, uterine stromal cells were induced for in vitro decidualization for 48 h. HB-EGF siRNA sequence was shown as follows: 5′-CCGUCUGUCUUCUUGUCAUTT and 5′-AUGACAAGAAGACAGACGGTT. The nonspecific scrambled siRNA served as negative control, and its sequence was described previously [13].

### 2.6. Cell Proliferation

After uterine stromal cells were seeded in 96-well plates and then treated with rHB-EGF for 24 h in the presence of estradiol-17*β* and progesterone, 20 *μ*l of MTS reagent (Promega, G3580) was added to each well and incubated at 37°C for 3 h. Absorbance was measured at 490 nm using a 96-well plate reader. Every experiment was performed in triplicate.

### 2.7. Cell Cycle Analysis

After synchronization, stromal cells were incubated with rHB-EGF for 24 h in the presence of estradiol-17*β* and progesterone. Then, cells were washed with PBS and harvested by trypsinization, centrifuged, and then fixed overnight at 4°C in 70% ethanol. The fixed cells were washed with PBS and stained with 0.5 ml PI/RNase staining buffer (BD Biosciences, 550825) for 20 min at room temperature. Then, the cells were analyzed by flow cytometry.

### 2.8. Cell Apoptosis Analysis

The Annexin V-FITC apoptosis detection kit (Beyotime, C1062) was used to test the apoptosis of stromal cells. Briefly, after treatment with NAC or rHB-EGF, stromal cells were exposed to H_2_O_2_ in the presence of estradiol-17*β* and progesterone and then harvested by trypsinization. Cells were resuspended with binding buffer along with the addition of Annexin V-FITC and PI. After gentle vortex, the mixture was incubated for 20 min in the dark and then analyzed by flow cytometry.

### 2.9. Caspase-3 Activity Measurement

Caspase-3 activity was determined by Caspase 3 Activity Assay Kit (Beyotime, C1116). After treatment as described above, stromal cells were lysed and centrifuged for 15 min. Supernatants were mixed with 10 *μ*l of Ac-DEVD-pNA substrate, and yellow pNA were measured at 405 nm using a 96-well plate reader.

### 2.10. Mitochondrial Membrane Potential Measurement

Mitochondrial membrane potential was measured by the corresponding assay kit with JC-1 (Beyotime, C2005). After treatment as described above, stromal cells were incubated with JC-1 staining solution (5 *μ*M) for 20 min and then washed twice with JC-1 buffer solution. The stained cells were analyzed by flow cytometry. The ratio of red and green fluorescent intensities indicated changes in the mitochondrial membrane potential.

### 2.11. Alkaline Phosphatase (ALP) Activity Assay

ALP activity was measured by alkaline phosphatase activity assay kit (Beyotime, P0321). After treatment as mentioned above, stromal cells were washed twice by PBS and then lysed with lysis buffer followed by the addition of pNPP substrate solution. Absorbance was measured at 405 nm using a 96-well plate reader.

### 2.12. Determination of ROS Level

After treatment as described above, stromal cells were incubated with fluorescent probe DCFH-DA (Beyotime, S0033, 20 *μ*M), dihydroethidium (Beyotime, S0063, 10 *μ*M), or MitoSOX™ Red mitochondrial superoxide indicator (Invitrogen, M36008, 5 *μ*M) at 37°C for different times, washed three times to remove redundant probe, and then analyzed by Multi-Detection Microplate Reader or flow cytometry to determine the levels of intracellular ROS, O_2_^−^, and mitochondrial O_2_^−^, respectively.

### 2.13. Determination of Malondialdehyde (MDA)

MDA content was determined by Lipid Peroxidation MDA Assay Kit (Beyotime, S0131). After proteins from stromal cells were extracted by lysis buffer and centrifuged, supernatants were blended with TBA detection solution, transferred to a 96-well plate to measure the absorbance at 532 nm, and then calculated MDA content according to standard curve.

### 2.14. Determination of Antioxidant Enzyme Activities

After stromal cells were lysed and centrifuged, supernatants were collected for analyzing the activities of superoxide dismutase (SOD), catalase (CAT), and glutathione peroxidase (GPX) by the corresponding assay kit (Beyotime, S0103, S0051, or S0058). For SOD activity, supernatants were incubated with WST-8/enzyme solution concomitant with an addition of reaction-started working solution, and then, the absorbance was measured at 450 nm to calculate SOD activity in accordance with the corresponding formula. For CAT activity, supernatants were mixed with 5 mM H_2_O_2_ for 5 min followed by a supplementation of substrate solution, and then, the absorbance was assessed at 520 nm to calculate CAT activity according to standard curve. For GPX activity, supernatants were mixed with working solution along with the addition of peroxide reagent and then determined the absorbance at 340  nm to calculate GPX activity in the light of the corresponding formula.

### 2.15. Measurement of NADPH Oxidase (NOX) Activity

NOX activity was determined by NOX Detection Assay Kit (Solarbio, BC0630). After supernatants were collected and mitochondria were disrupted by freezing and thawing, samples were mixed with reaction solution containing 2,6-dichloroindophenol indophenol (DCPIP) and then the absorbance was measured at 600 nm to determine the activity of NOX in accordance with the corresponding calculation formula.

### 2.16. Measurement of Glutathione Content

Intracellular reduced glutathione (GSH) and oxidized glutathione (GSSG) contents were determined by GSH and GSSG Assay Kits (Beyotime, S0052). After 10 *μ*l of supernatants were mixed with 50 *μ*l NADPH (0.5 mg/ml) and 150 *μ*l substrate solution, absorbance was read at 412 nm every 5 min for a total of 25 min. Then, GSH and GSSG contents were calculated according to standard curve.

### 2.17. Measurement of Intracellular Adenosine Triphosphate (ATP)

Intracellular ATP level was determined by using Enhanced ATP Assay Kit (Beyotime, S0027). Stromal cells were lysed using lysis buffer and centrifuged for 5 min to collect the supernatants. Then, luminescence was measured after addition of detection solution and supernatants, and ATP level was calculated according to standard curve.

### 2.18. Measurement of Activities of Mitochondrial Respiratory Chain Complex I, II, and III

Activities of mitochondrial respiratory chain complex I, II, and III were assessed by the corresponding assay kit (Solarbio, BC0510, BC3230, or BC3240). After addition of extracting solution, supernatants were collected, and mitochondria were disrupted by freezing and thawing. These samples were then mixed with different reaction solutions containing NADH, DCPIP, or cytochrome C to determine the activities of mitochondrial respiratory chain complex I, II, and III by measuring the absorbance at 340, 605, or 550 nm in accordance with the corresponding calculation formula.

### 2.19. Statistics

All experiments were independently repeated at least three times. Significance of difference between two groups was compared by the independent samples *t*-test. The multiple comparisons were tested with one-way ANOVA. The differences were considered significant at *P* < 0.05. All statistical analyses were performed using SPSS19.0 software program (SPSS Inc., Chicago).

## 3. Results

### 3.1. Effects of HB-EGF on the Proliferation and Differentiation of Uterine Stromal Cells

**Figure 1:**
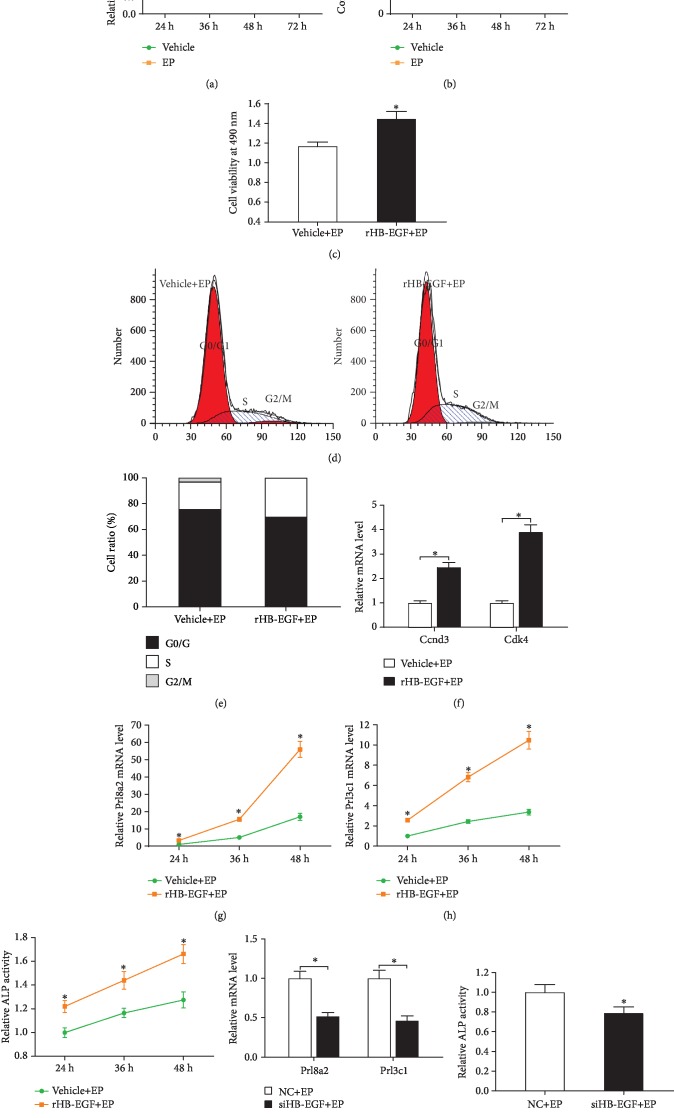
Effects of HB-EGF on the proliferation and differentiation of uterine stromal cells during in vitro decidualization. (a) Real-time PCR analysis of HB-EGF mRNA expression after treatment with estrogen and progesterone for 24, 36, 48, and 72 h. (b) ELISA analysis of HB-EGF protein after treatment with estrogen and progesterone. (c) Effects of HB-EGF on stromal cell proliferation. After treatment with rHB-EGF for 24 h in the presence of estrogen and progesterone, stromal cells were analyzed by MTS assay. (d and e) Flow cytometry analysis of HB-EGF role in cell cycle of stromal cells. (f) Effects of HB-EGF on the expression of Ccnd3 and Cdk4 in stromal cells. (g–i) Effects of HB-EGF on Prl8a2 and Prl3c1 expression as well as ALP activity. (j and k) Effects of HB-EGF siRNA on Prl8a2 and Prl3c1 expression as well as ALP activity. EP: estrogen plus progesterone; NC: negative control; siHB-EGF: HB-EGF siRNA. Data are shown as mean ± SEM. Asterisks denote significance (*P* < 0.05).

HB-EGF mRNA was abundant in the decidualing stromal cells, and its expression was gradually increased as decidualization progress, reaching the highest level at 72 h after treatment with estrogen and progesterone (Figure 1(a)). Consistently, further analysis of HB-EGF protein by ELISA also revealed a time-dependent increase after induction for in vitro decidualization (Figure 1(b)). Replenishment of rHB-EGF, which led to an obvious enhancement in HB-EGF protein content but did no effect its mRNA level as well as stromal cell morphology, enhanced the proliferation activity of stromal cells and induced the accumulation of cells in S phase with the simultaneous reduction in the proportion of cells in G0/G1 and G2/M phases (Figures 1(c)–1(e); Supplementary [Supplementary-material supplementary-material-1]; Supplementary Figures [Supplementary-material supplementary-material-1]A and [Supplementary-material supplementary-material-1]B). In the meantime, rHB-EGF elevated the expression of cyclin D3 (Ccnd3) and cyclin-dependent kinase 4 (Cdk4) (Figure 1(f)).

To further elucidate the effects of HB-EGF on stromal cell differentiation, we investigated its effects on the expression of prolactin family 8, subfamily a, member 2 (Prl8a2), prolactin family 3, subfamily c, member 1 (Prl3c1), and activity of alkaline phosphatase (ALP), which are well-established stromal differentiation markers during decidualization [13, 14]. The results showed that rHB-EGF markedly upregulated the expression of Prl8a2 and Prl3c1 and promoted ALP activity in a time-dependent manner with the highest level at 48 h (Figures 1(g)–1(i)). In contrast, transfection with HB-EGF siRNA, which efficiently reduced this corresponding mRNA and protein levels, could dramatically hamper the expression of Prl8a2 and Prl3c1 mRNA and reduce ALP activity (Figures 1(j) and 1(k); Supplementary Figures [Supplementary-material supplementary-material-1]C and [Supplementary-material supplementary-material-1]D).

### 3.2. HB-EGF Protected Uterine Stromal Cell Differentiation against H_2_O_2_-Induced Oxidative Damage

**Figure 2:**
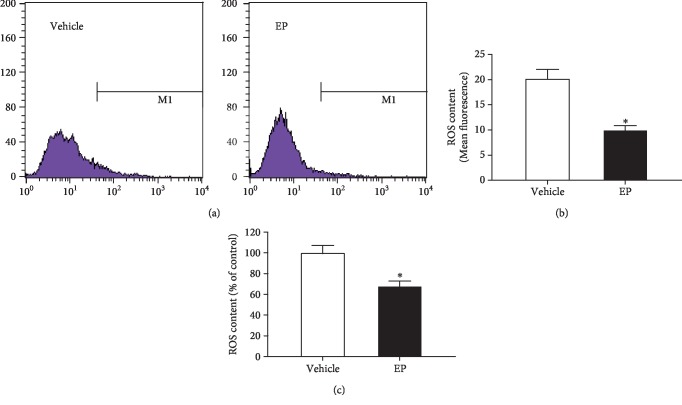
Intracellular ROS level during in vitro decidualization. (a and b) Flow cytometry analysis of ROS level in uterine stromal cells with/without estrogen and progesterone. (c) Intracellular ROS was detected by Multi-Detection Microplate Reader after treatment with estrogen and progesterone.

**Figure 3:**
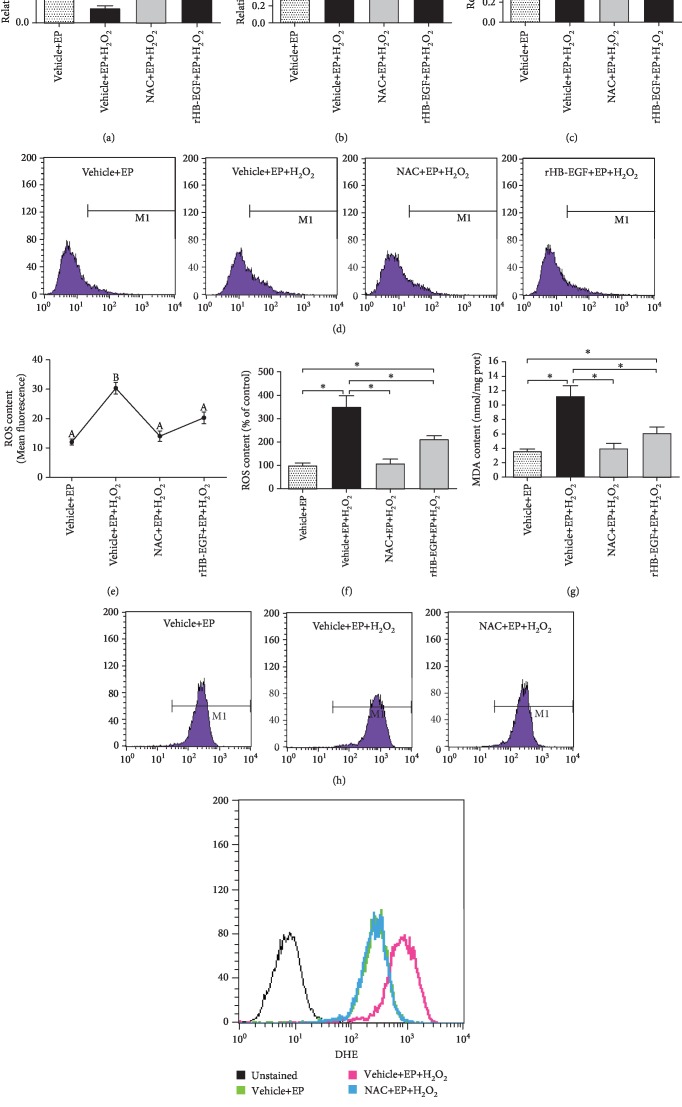
HB-EGF protects stromal cell differentiation against H_2_O_2_-induced oxidative damage during in vitro decidualization. (a–c) NAC and HB-EGF abrogated the repression of H_2_O_2_ on Prl8a2 and Prl3c1 expression as well as ALP activity. After treatment with NAC or rHB-EGF and then addition of H_2_O_2_, Prl8a2 and Prl3c1 expression and ALP activity were determined in the presence of estrogen and progesterone. (d–f) NAC and HB-EGF weakened the induction of H_2_O_2_ on intracellular ROS level. After treatment with NAC or rHB-EGF and then addition of H_2_O_2_, intracellular ROS levels were detected by flow cytometry or Multi-Detection Microplate Reader in the presence of estrogen and progesterone. (g) NAC and HB-EGF impeded the induction of H_2_O_2_ on MDA content. (h and i) NAC blocked the induction of H_2_O_2_ on intracellular O_2_^−^ level. Bars with different letters at the top differ significantly.

**Figure 4:**
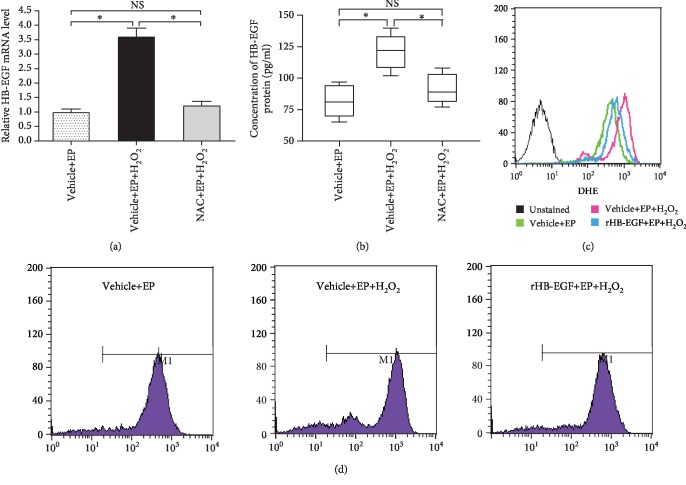
Effects of HB-EGF on intracellular O_2_^−^ level in H_2_O_2_-treated stromal cells. (a) Real-time PCR analysis of HB-EGF mRNA expression after treatment with H_2_O_2_ or both H_2_O_2_ and NAC. (b) ELISA analysis of HB-EGF protein level after treatment with H_2_O_2_ or both H_2_O_2_ and NAC. (c and d) HB-EGF hindered the induction of H_2_O_2_ on intracellular O_2_^−^ level. After treatment with rHB-EGF and then exposure to H_2_O_2_, the intracellular O_2_^−^ level was analyzed by flow cytometry during in vitro decidualization. NS: not significant.

After stromal cells were subjected to in vitro decidualization, intracellular ROS level was significantly reduced compared with control (Figures 2(a)–2(c)), implying that a low level of ROS may be beneficial for uterine decidualization. When exposed to H_2_O_2_ in the presence of estrogen and progesterone, stromal cell differentiation exhibited an obvious impairment as evidenced by the reduced expression or activity of Prl8a2, Prl3c1, and ALP, whereas intracellular ROS and O_2_^−^ levels were remarkably raised along with the elevated content of malondialdehyde (MDA), which was regarded as a biomarker for oxidative stress (Figures 3(a)–3(i)) [15]. But this impairment was ameliorated by ROS scavenger NAC which efficiently cleared up the intracellular levels of ROS and O_2_^−^ and weakened the upregulation of MDA level elicited by H_2_O_2_ (Figures 3(a)–3(i)). Further analysis found that HB-EGF mRNA and protein levels were significantly enhanced in oxidative stress-mediated stromal differentiation damage, but this enhancement was abrogated by NAC (Figures 4(a) and 4(b)). Extraneous rHB-EGF reversed the expression of Prl8a2 and Prl3c1 and restored ALP activity in H_2_O_2_-treated stromal cells undergoing decidualization followed by a decline in the levels of ROS, O_2_^−^, and MDA (Figures 3(a)–3(g); Figures 4(c) and 4(d)).

### 3.3. HB-EGF Rescued Antioxidant Enzyme Activities in H_2_O_2_-Treated Uterine Stromal Cells

**Figure 5:**
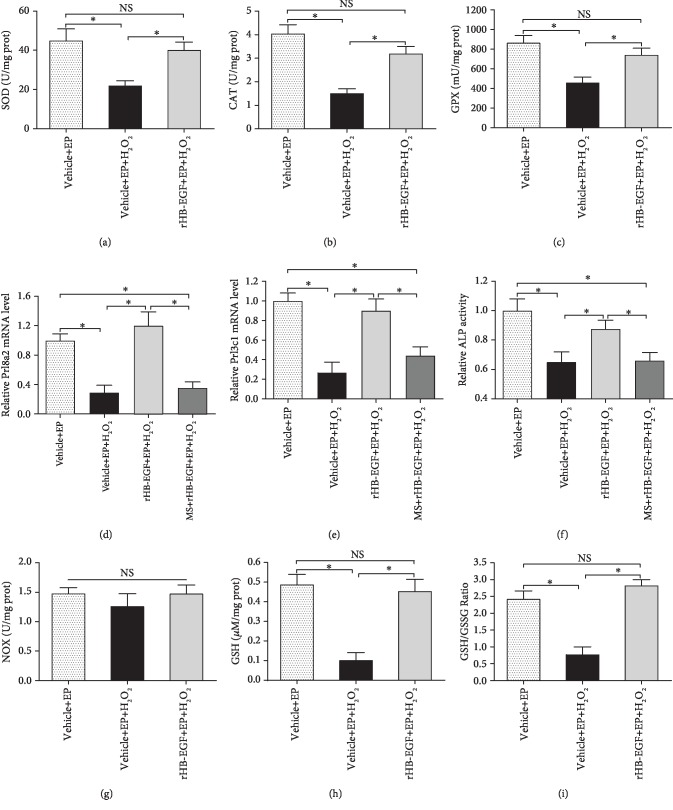
HB-EGF rescues antioxidant capacity in H_2_O_2_-treated stromal cells. (a–c) HB-EGF restored the activities of antioxidant enzymes (a) SOD, (b) CAT, and (c) GPX after exposure to H_2_O_2_. (d–f) GPX inhibitor MS impeded the induction of HB-EGF on Prl8a2 and Prl3c1 expression as well as ALP activity under oxidative stress. (g) HB-EGF had no effect on NOX activity in stromal cells under oxidative stress. (h and i) HB-EGF rescued the content of GSH in H_2_O_2_-induced stromal cells and restored the ratio of GSH/GSSG in H_2_O_2_-induced stromal cells.

To elucidate the mechanism by which HB-EGF enhanced the oxidation resistance of decidual stromal cells, we examined its effects on the activities of antioxidant enzymes SOD, CAT, and GPX. As shown in Figures 5(a)–5(c), the activities of SOD, CAT, and GPX were significantly decreased after exposure to H_2_O_2_, while addition of rHB-EGF recovered the activities of the above antioxidant enzymes. Furthermore, GPX inhibitor MS completely attenuated the protective effects of rHB-EGF on the expression or activity of Prl8a2, Prl3c1, and ALP in H_2_O_2_-treated uterine stromal cells in the presence of estrogen and progesterone (Figures 5(d)–5(f)).

### 3.4. Effects of HB-EGF on NOX Activity and GSH Content in H_2_O_2_-Treated Uterine Stromal Cells

Because NOX is one of the major sources of cellular ROS [16], we assessed the effects of HB-EGF on NOX activity. After exposure to H_2_O_2_, no distinguishable difference in NOX activity was detected in the absence or presence of rHB-EGF (Figure 5(g)). It has been previously reported that GSH acts as a key cellular antioxidant to maintain the balance of redox state [5]. Addition of rHB-EGF rescued the content of GSH in H_2_O_2_-induced stromal cells and restored the ratio of GSH/GSSG (Figures 5(h) and 5(i)).

### 3.5. HB-EGF Protected Mitochondrial Function in H_2_O_2_-Treated Uterine Stromal Cells

**Figure 6:**
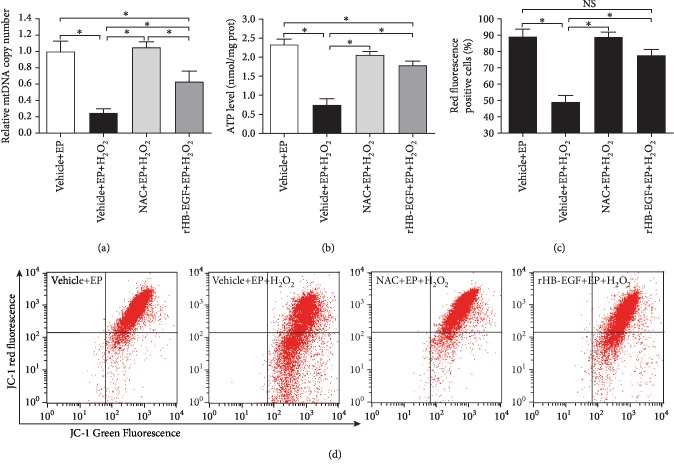
HB-EGF prevents mitochondrial dysfunction in H_2_O_2_-treated stromal cells. (a) NAC and HB-EGF impeded the impairment of H_2_O_2_ on mtDNA copy number in stromal cells during in vitro decidualization. After treatment with NAC or rHB-EGF and then addition of H_2_O_2_, mtDNA copy number was determined by real-time PCR. (b) NAC and HB-EGF blocked the impairment of H_2_O_2_ on ATP level in stromal cells during in vitro decidualization. (c and d) Flow cytometry analysis of mitochondrial membrane potential after treatment with NAC or rHB-EGF and then addition of H_2_O_2_ during in vitro decidualization.

**Figure 7:**
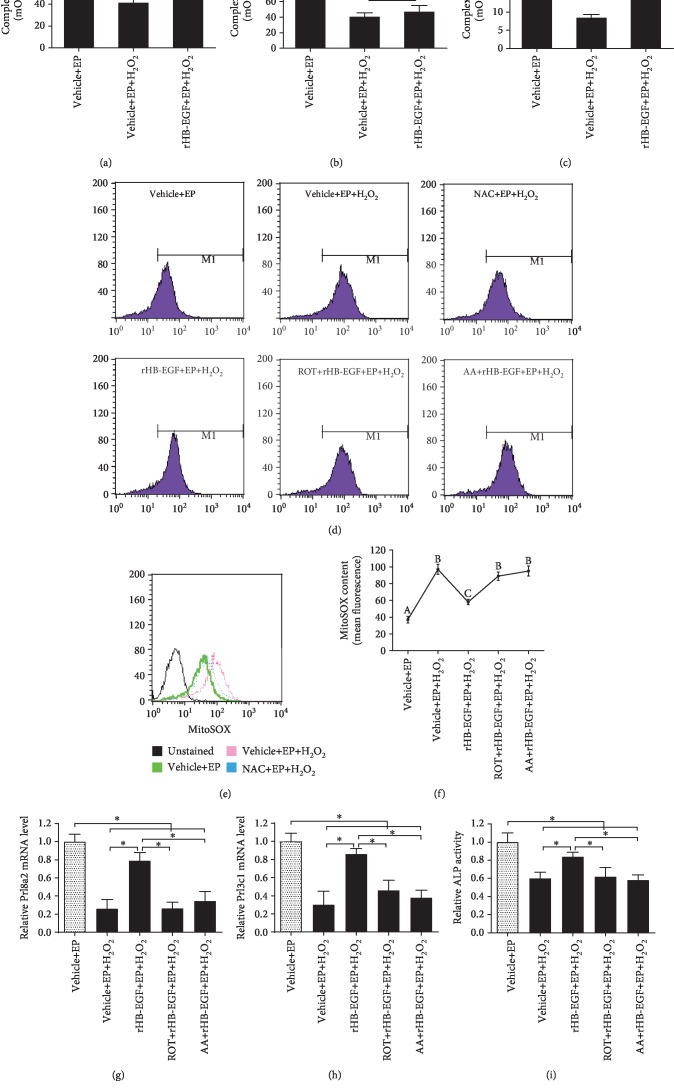
Effects of HB-EGF on mitochondrial O_2_^−^ and mitochondrial respiratory chain complex under oxidative stress. (a–c) Effects of HB-EGF on the activities of mitochondrial respiratory chain complex I, II, and III in stromal cells under oxidative stress. (d and e) NAC reduced the mitochondrial O_2_^−^ in stromal cells after exposure to H_2_O_2_. (d and f) Mitochondrial respiratory chain complex I and III mediated the effects of HB-EGF on mitochondrial O_2_^−^ under oxidative stress. After treatment with ROT or AA and addition of rHB-EGF, mitochondrial O_2_^−^ was analyzed by flow cytometry under oxidative stress. (g–i) Mitochondrial respiratory chain complex I and III mediated the regulation of HB-EGF on Prl8a2, Prl3c1, and ALP. After treatment with ROT or AA followed by the addition of rHB-EGF, Prl8a2 and Prl3c1 expression and ALP activity were determined.

To explore the protective role of HB-EGF in mitochondrial function, we first analyzed its influence on mitochondrial DNA (mtDNA) copy number and ATP level. After treatment with H_2_O_2_, mtDNA copy number and ATP level were distinctly lessened, but this decline was abrogated by ROS scavenger NAC (Figures 6(a) and 6(b)). Consistent with the above result, rHB-EGF partially prevented the impairment of mtDNA copy number and ATP level elicited by H_2_O_2_ (Figures 6(a) and 6(b)). Afterward, the effects of HB-EGF on mitochondrial membrane potential were assessed by JC-1 fluorescent probe. Under oxidative stress, mitochondrial membrane potential was drastically diminished, whereas addition of NAC or rHB-EGF reversed this effectiveness (Figures 6(c) and 6(d)). Further study found that H_2_O_2_ markedly suppressed the activities of mitochondrial respiratory chain complex I, II, and III, while rHB-EGF obviously rescued the activities of mitochondrial respiratory chain complex I and III but did not restore the activity of mitochondrial respiratory chain complex II (Figures 7(a)–7(c)) After treatment with H_2_O_2_, mitochondrial O_2_^−^ level was prominently boosted, but this elevation was weakened by NAC and rHB-EGF (Figures 7(d)–7(f)). Addition of mitochondrial respiratory chain complex I inhibitor ROT or mitochondrial respiratory chain complex III inhibitor AA could efficiently impede the repression of mitochondrial O_2_^−^ production by rHB-EGF and weakened the stimulation of rHB-EGF on the expression of Prl8a2 and Prl3c1 as well as ALP activity (Figures 7(d), 7(f)–7(i)).

### 3.6. HB-EGF Prevented Apoptosis of Uterine Stromal Cells after Exposure to H_2_O_2_

**Figure 8:**
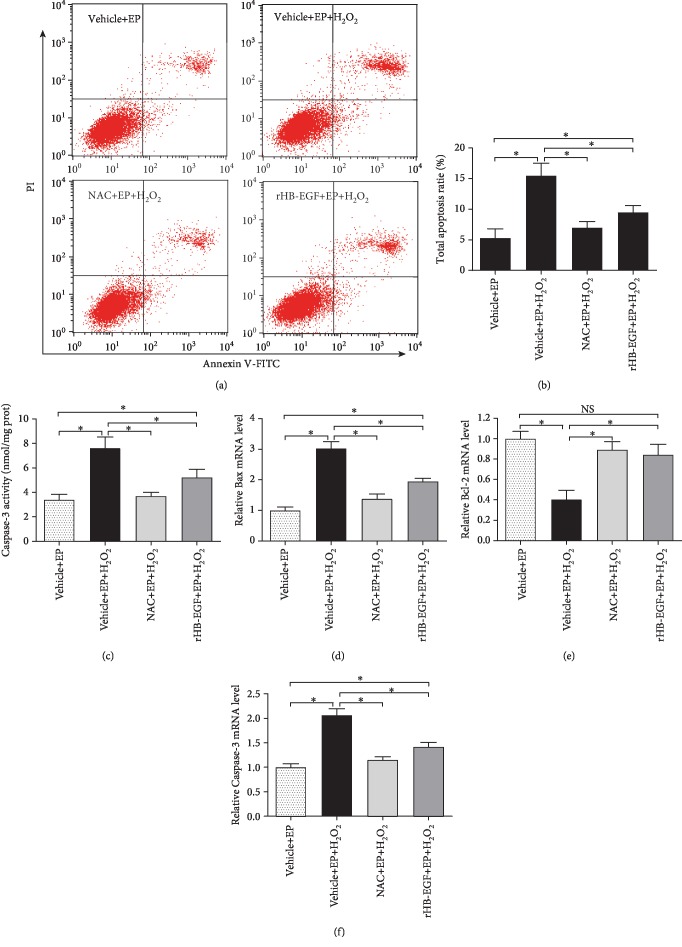
HB-EGF prevents the apoptosis of uterine stromal cells after exposure to H_2_O_2_. (a and b) NAC and HB-EGF blocked the induction of H_2_O_2_ on stromal cell apoptosis. After treatment with NAC or rHB-EGF and then addition of H_2_O_2_, cell apoptosis was determined by flow cytometry. (c) NAC and HB-EGF blocked the stimulation of H_2_O_2_ on Caspase-3 activity. (d) NAC and HB-EGF hindered the stimulation of H_2_O_2_ on Bax mRNA expression. (e) NAC and HB-EGF canceled the repression of H_2_O_2_ on Bcl-2 mRNA expression. (f) NAC and HB-EGF weakened the stimulation of H_2_O_2_ on Caspase-3 mRNA expression.

It is well-known that a variety of key events in apoptosis focus on mitochondria [17]. As stated above, HB-EGF might prevent mitochondrial dysfunction after exposure to H_2_O_2_, suggesting the antiapoptotic role of HB-EGF in uterine stromal cells (Figures 8(a) and 8(b)). As expected, rHB-EGF partially hampered the induction of stromal cell apoptosis by H_2_O_2_ (Figures 8(a) and 8(b)). The similar result was also observed after treatment with ROS scavenger NAC (Figures 8(a) and 8(b)). To further clarify the molecular basis for the antiapoptotic role of HB-EGF, we examined its regulation on Caspase-3, Bax, and Bcl-2 which were important for cell apoptosis [18]. After exposure to H_2_O_2_, Caspase-3 activity was notably enhanced, but this enhancement was hindered by NAC and rHB-EGF (Figure 8(c)). Consistently, rHB-EGF and NAC attenuated the expression of Bax and Caspase-3 mRNA under oxidative stress and recovered the level of Bcl-2 mRNA (Figures 8(d)–8(f)).

## 4. Discussion

Although HB-EGF is required for uterine decidualization, its antioxidant role remains largely unclear. The present study examined the effects of HB-EGF on the proliferation and differentiation of uterine stromal cells and explored whether HB-EGF might protect stromal differentiation from oxidative stress. Here, we found that HB-EGF promoted the proliferation of stromal cells along with the accelerated transition of cell cycle from G1 into S phase and enhanced the expression or activity of Prl8a2, Prl3c1, and ALP which are well-established markers for uterine stromal cell differentiation during decidualization [13, 14], further reinforcing the role of HB-EGF in uterine decidualization. Further analysis evidenced that exogenous rHB-EGF upregulated the expression of Ccnd3 which was a G1 phase cell cycle regulator and involved in stromal cell proliferation and differentiation [19]. Adenoviral delivery of antisense Ccnd3 abrogated the induction of stromal cell differentiation by HB-EGF [10]. Together, these observations indicate that Ccnd3 is a downstream of HB-EGF in uterine decidualization.

When ROS generation exceeds the scavenging capacity by antioxidants as a result of overabundant ROS, oxidative stress occurs and negatively impacts reproductive processes [5, 20]. H_2_O_2_ has been extensively used to induce oxidative stress which is characterized by the elevated levels of intracellular ROS and MDA [15, 21]. During in vitro decidualization, H_2_O_2_ impaired stromal cell differentiation, but this impairment was abrogated by ROS scavenger NAC. Previous studies found that abundant HB-EGF was noted in liver cancer cells, aortic smooth muscle cells, and retinal pigment and gastric epithelial cells after exposure to H_2_O_2_ [22–25]. The same result was also observed in uterine stromal cells. Addition of rHB-EGF attenuated the elevated levels of intracellular ROS, O_2_^−^, and MDA elicited by H_2_O_2_ and restored stromal cell differentiation, demonstrating an antioxidant function of HB-EGF in oxidative stress, which was further reinforced by the observation that HB-EGF might decrease the production of intestinal ROS in the ischemia/reperfusion injury model [12]. Further analysis evidenced that HB-EGF enhanced the activity of antioxidant enzyme SOD which was a catalyst for dismutation of O_2_^−^ to H_2_O_2_ [26]. In the meantime, CAT and GPX could catalyze H_2_O_2_ conversion to water [4]. Under oxidative stress, HB-EGF heightened the activity of CAT and GPX. Simultaneously, as a natural nonenzymatic antioxidant, GSH performs considerable effort in maintaining the balance of redox state [5]. The oxidation of GSH to GSSG and subsequent decrease in the GSH/GSSG ratio was associated with oxidative stress [27]. During in vitro decidualization, HB-EGF increased GSH content and GSH/GSSG ratio in H_2_O_2_-treated stromal cells. Taken together, these results reveal that HB-EGF may exert its antioxidant function in oxidative stress-mediated uterine decidualization damage through the elevated antioxidant capacity.

Mitochondria are important intracellular organelles for ATP synthesis and ROS generation which are two considerable indicators of mitochondrial physiology [4, 5]. Under oxidative stress, HB-EGF improved the defects of ATP content followed by the reduction of mitochondrial O_2_^−^ level, revealing a role of HB-EGF in preventing mitochondrial dysfunction. Due to the lack of protection from histones, mitochondria are prone to oxidative injury by excessive ROS, resulting in mtDNA impairment which reduces the enzymatic activities of mitochondrial respiratory chain complex [4, 5]. After exposure to H_2_O_2_, HB-EGF restored the faults of mtDNA copy number and mitochondrial respiratory chain complex I and III activities that might reflect the integrity of mitochondrial function [28, 29]. Moreover, replenishment of mitochondrial respiratory chain complex I and III inhibitor ROT and AA weakened the stimulation of rHB-EGF on the expression of Prl8a2 and Prl3c1 as well as ALP activity. Meanwhile, mitochondrial membrane potential is essential for normal mitochondrial function [30]. HB-EGF ameliorated the damage of oxidative stress on mitochondrial membrane potential. Collectively, these observations imply that HB-EGF plays an important role in improving mitochondrial dysregulation.

Previous evidences have reported that mitochondrial dysfunction resulted in cell apoptosis [4, 31]. Compared to undifferentiated endometrial stromal cells, decidual cells apparently conferred resistance to oxidative cell death which was necessary for the establishment of successful decidualization [8, 32]. During in vitro decidualization, HB-EGF might ameliorate oxidative stress-induced stromal cell apoptosis. It is well-established that cell apoptosis is controlled by proapoptotic Bax, anti-apoptotic Bcl-2, and Caspase-3 which is the executor of apoptosis [18, 33]. Addition of rHB-EGF could attenuate the elevation of Bax and Caspase-3 under oxidative stress and restore the level of Bcl-2 mRNA, further highlighting the antiapoptotic role for HB-EGF.

**Figure 9:**
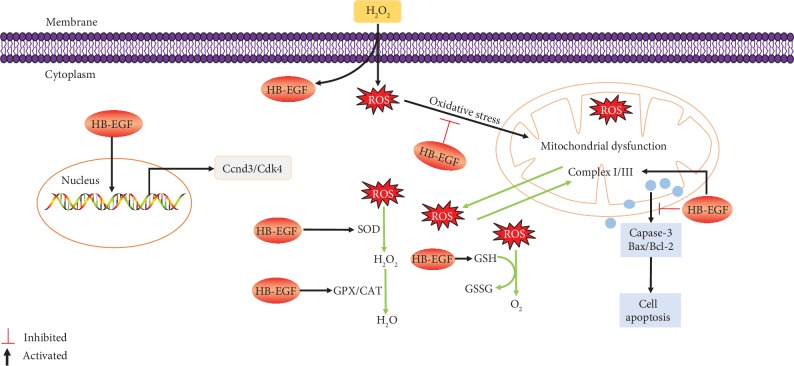
Schematic diagram summarizes the protective effects of HB-EGF on oxidative stress-mediated uterine decidualization damage. HB-EGF might induce proliferation and differentiation of uterine stromal cells through targeting Ccnd3 and Cdk4 and prevent the impairment of oxidative stress on uterine decidualization by improving the antioxidant capacity of SOD, CAT, GPX, and GSH and restoring the mitochondrial function. Meanwhile, HB-EGF ameliorated oxidative stress-induced stromal cell apoptosis via Caspase-3, Bax, and Bcl-2.

In conclusion, HB-EGF may protect uterine decidualization against oxidative stress by improving antioxidant capacity, restoring mitochondrial function, and inhibiting cell apoptosis (Figure 9).

## Figures and Tables

**Table 1:** Primers for real-time PCR.

Gene	Forward primer	Reverse primer
HB-EGF	GGCTGTAGTACTGTCGTCCG	GTCCTCCTCAGTGGGAGCTA
Ccnd3	CCTCCTACTTCCAGTGCGTG	GGCAGACGGTACCTAGAAGC
Cdk4	GTGGCTGAAATTGGTGTCGG	TAACAAGGCCACCTCACGAA
Prl8a2	AGCCAGAAATCACTGCCACT	TGATCCATGCACCCATAAAA
Prl3c1	GCCACACGATATGACCGGAA	GGTTTGGCACATCTTGGTGTT
mtDNA	CGATTCTTTACCTTTCACTTCATCTT	GAGGGCGTCTTTGATTGTGT
Bax	CCGGCGAATTGGAGATGAACT	CCAGCCCATGATGGTTCTGAT
Bcl-2	TCAGAGCGAGAAGGTAGGGA	CTGTGGGGTAACAAGAAGGTC
Caspase-3	CTGGACTGTGGCATTGAGAC	GCAAAGGGACTGGATGAACC
Gapdh	GCCTTCCGTGTTCCTACCC	TGCCTGCTTCACCACCTTC

## Data Availability

The data used to support the findings of this study are included within the article.

## Supplementary Materials

Supplementary 1 Figure 1: H&E staining analysis of stromal cell morphology after treatment with rHB-EGF during in vitro decidualization. After treatment with rHB-EGF for 48 h in the presence of estrogen and progesterone, stromal cells in coverslip were fixed with 4% cold paraformaldehyde, washed with PBS, and stained with Hematoxylin-Eosin (H&E). Bar = 200 *μ*m.

Supplementary 2 Figure 2: effects of rHB-EGF and HB-EGF siRNA on its mRNA or protein levels during in vitro decidualization. (A) Real-time PCR analysis of HB-EGF mRNA expression after treatment with rHB-EGF. (B) ELISA analysis of HB-EGF protein level after treatment with rHB-EGF. (C) Real-time PCR analysis of HB-EGF mRNA expression after transfection with HB-EGF siRNA. (D) ELISA analysis of HB-EGF protein level after transfection with HB-EGF siRNA.
